# The Possible Effect of β-Blocker Use on the Circulating MMP-2/TIMP-2 System in Patients with Chronic Kidney Disease on Conservative Treatment

**DOI:** 10.3390/jcm13071847

**Published:** 2024-03-23

**Authors:** Magdalena Kopańko, Magdalena Zabłudowska, Dariusz Pawlak, Beata Sieklucka, Anna Krupa, Katarzyna Sokołowska, Marta Ziemińska, Krystyna Pawlak

**Affiliations:** 1 Department of Monitored Pharmacotherapy, Medical University of Bialystok, Mickiewicza 2C, 15-222 Bialystok, Poland; 38599@student.umb.edu.pl (M.K.); 38622@student.umb.edu.pl (M.Z.); beata.sieklucka@umb.edu.pl (B.S.); katarzyna.chilkiewicz@umb.edu.pl (K.S.); marta.zieminska26@gmail.com (M.Z.); 2 Department of Pharmacodynamics, Medical University of Bialystok, Mickiewicza 2C, 15-222 Bialystok, Poland; dariusz.pawlak@umb.edu.pl; 3 Department of Internal Medicine and Metabolic Diseases, Medical University of Bialystok, M. Sklodowskiej-Curie 24A, 15-276 Bialystok, Poland; anna.krupa@sd.umb.edu.pl

**Keywords:** β-blockers, chronic kidney disease (CKD), metalloproteinase 2 (MMP-2), tissue inhibitor of metalloproteinase 2 (TIMP-2), inflammation, oxidative stress

## Abstract

**Background**: The purpose of the study was to determine whether the use of β-adrenoceptor antagonists (β-blockers) can affect metalloproteinase 2 (MMP-2) and its tissue inhibitor (TIMP-2) in patients with chronic kidney disease (CKD) on conservative treatment. **Methods**: The circulating MMP-2/TIMP-2 system, proinflammatory cytokines (tumor necrosis factor-alpha (TNF-α) and interleukin-6 (IL-6), and the marker of oxidative stress—Cu/Zn superoxide dismutase (Cu/Zn SOD)—were measured in 23 CKD patients treated with β-blockers [β-blockers (+)] and in 27 CKD patients not receiving the above medication [β-blockers (−)]. **Results**: The levels of MMP-2, TIMP-2, and IL-6 were significantly lower in the β-blockers (+) than in the β-blockers (−) group, whereas Cu/Zn SOD concentrations were not affected by β-blocker use. There was a strong, independent association between MMP-2 and TIMP-2 in both analyzed patient groups. In the β-blockers (+) group, MMP-2 levels were indirectly related to the signs of inflammation, whereas in the β-blockers (−) group, the alterations in the MMP-2/TIMP-2 system were associated with the oxidative stress marker and CKD etiology. **Conclusions**: This study is the first to suggest that the use of β-blockers was associated with the reduction in IL-6 and the MMP-2/TIMP-2 system in CKD, providing a pharmacological rationale for the use of β-blockers to reduce inflammation and abnormal vascular remodeling in CKD.

## 1. Introduction

Hypertension and chronic kidney disease (CKD) are pathophysiological states that are intimately related, as hypertension can be both a cause and a consequence of CKD. The prevalence of hypertension is high among patients with CKD, and about 60% of the population requires treatment with three or more antihypertensive drugs, indicating that resistant hypertension is common in CKD. Thus, the early diagnosis as well as proper treatment of hypertension plays a central role in the management of CKD [1,2]. Additionally, achieving optimal blood pressure (BP) is associated with reduced renal and cardiovascular risk in this population [3]. 

According to the current standards of hypertension treatment in CKD, β-adrenoceptor antagonists (β-blockers), though not recommended as first-line therapy, may be useful nonetheless for the treatment of hypertension in CKD patients with specific indications [4]. However, increased sympathetic activity is known to be a contributor to hypertension in CKD, and it is associated with an increased risk of cardiovascular events and renal disease progression [5]. β-blockers used to treat hypertension also block the β1-adrenergic receptors expressed primarily in cardiac tissue, reducing the effect of catecholamine on heart rate and cardiac contractility. Although the precise mechanism leading to a long-term reduction in systemic BP remains unclear, a reduction in systemic vascular resistance is probably responsible for the antihypertensive effect of β-blockers [6]. The additional vasodilatory mechanisms expressed by individual β-blockers include the release of nitric oxide (NO) or the blockade of α-adrenoceptors [7].

Apart from antihypertensive effects, treatment with several β-blockers was associated with a reduced severity of systemic inflammation both in clinical [8] and experimental studies [9,10]. The anti-inflammatory effect of β-blockers included a decrease in proinflammatory cytokines, like interleukin (IL) 6 (IL-6), IL-1β [9], and tumor necrosis factor-α (TNF-α) [10], and an increase in the levels of anti-inflammatory cytokine IL-10 [9,10]. Moreover, previous studies suggest that some β-blockers, like nebivolol, metoprolol, and carvedilol, can exert antioxidant effects [11,12,13,14,15,16]. The above features of β-blockers could explain their therapeutic benefits in the cardiovascular system. 

Matrix metalloproteinase 2 (MMP-2) is a zinc-containing endopeptidase that is involved in the proteolytic degradation of extracellular matrix (ECM) and non-ECM substrates, like cell adhesion molecules, growth factors, and their receptors [17]. The major modulator of MMP-2 activity is its endogenous inhibitor—TIMP-2—and the imbalance between MMP-2 and TIMP-2 plays an important role in pathological tissue remodeling [18,19]. The results of some experimental studies suggest that β-blockers can affect metalloproteinases by reducing oxidative stress [13,14,16].

Our previous studies demonstrated increased circulating MMP-2 and TIMP-2 levels in patients with CKD, which was associated with hypercoagulability, oxidative stress, inflammation, carotid atherosclerosis, and cardiovascular risk [20,21,22,23]. Although some observational studies and single randomized trials showed that the use of β-blockers in CKD patients was associated with the reduction in cardiovascular events and improvement of survival [24,25,26,27,28], the class effect of this group on metalloproteinases/their inhibitors in CKD is unknown.

The present study was undertaken to determine whether the use of β-blockers can affect the MMP-2/TIMP-2 system in CKD patients on conservative treatment. Moreover, we wanted to establish the clinical and biochemical parameters that may influence the systemic MMP-2 and TIMP-2 levels both in β-blocker-treated [β-blockers (+)] and untreated [β-blockers (−)] patients with CKD.

## 2. Materials and Methods

### 2.1. Study Participants

The study involved 50 clinically stable patients with CKD (29 males and 21 females), who were treated conservatively at the Department of Nephrology and Clinical Transplantation, Medical University of Bialystok. All of them were in good general condition and free of active infection at the time of the study. None of the patients received immunosuppressive treatment; non-steroidal anti-inflammatory drugs; antioxidants such as Vitamin C, Vitamin E, or allopurinol; or recombinant human erythropoietin at least 3 months preceding the study. Before starting the study, 42% of the patients had previously been diagnosed with cardiovascular disease (CVD), based on prior ECGs and/or exercise tests, or one or more of typical clinical symptoms. Nine subjects (18%) were smokers. The pharmacological treatment of hypertension was used in 44 subjects (88%), which was based on the therapy of angiotensin-converting enzyme inhibitors (ACEI) or sartans, calcium channel antagonists (CCA), β-adrenoceptor antagonists, α-adrenoceptor antagonists, and diuretics. Moreover, 9 patients (18%) obtained statin. The estimated glomerular filtration rate (eGFR) values were calculated using the Chronic Kidney Disease Epidemiology Collaboration (CKD-EPI) equation and expressed in mL/min/1.73 m^2^ [29]. None of the patients underwent hemodialysis.

Twenty healthy volunteers matched for gender (10 men and 10 women) and age (average age 50.35 ± 16.82 years old) with patients with CKD served as controls for the determination of serum proinflammatory cytokines (TNF-α, IL-6), the marker of oxidative stress—Cu/Zn SOD, and the MMP-2/TIMP-2 system. All were on a regular diet and did not have any history of cardiovascular disease (CVD), hypertension, diabetes, or kidney disease (9% of people from the control group were smokers). 

### 2.2. Laboratory Methods

Blood samples from CKD patients and controls were taken from the antecubital vein in the morning, under fasting conditions. Citrated plasma and serum samples were prepared conventionally, aliquoted, and stored at −30 °C until the assay.

Serum levels of MMP-2 and plasma levels of TIMP-2 were measured by an enzyme-linked immunosorbent assay (ELISA) using commercially available standard kits (Human/mouse MMP-2 (total) and Human TIMP-2; Research and Diagnostic Systems Ltd., Abington, UK). Plasma Cu/Zn superoxide dismutase (Cu/Zn SOD), IL-6, and TNF-α concentrations were determined by human Cu/Zn SOD ELISA, human IL-6 HS ELISA, and human TNF-α ELISA kits from Bender MedSystems GmbH, Vienna, Austria. The hematological and biochemical parameters were determined by routine techniques using an automated analyzer (Cobas Mira, Roche Diagnostics, Rotkreus, Switzerland).

### 2.3. Statistical Analysis

The normality of distribution was tested using the Shapiro–Wilk test. The normally distributed data were expressed as mean ± SD. The non-Gaussian data were presented as a median (interquartile range). The chi-square test was used to compare frequency distributions. The Student’s *t*-test or nonparametric Mann–Whitney U test was used to compare differences between CKD subgroups, whereas the analysis of variance (ANOVA) was used to check differences between controls and the CKD subgroups. Univariate correlations between the variables were calculated by the Spearman rank correlation and quasi-Newton and Rosenbrock’s regression analysis when appropriate. A stepwise multiple regression analysis was performed to determine which variables could predict the MMP-2/TIMP-2 system in the β-blockers (+) and β-blockers (−) groups. A two-tailed *p* value < 0.05 was considered to be statistically significant. All analyses were performed using the Statistica v. 13 software (StatSoft, Tulsa, OK, USA). A graphic design presentation of results was prepared using GraphPad Prism 6 (GraphPad Software, La Jolla, CA, USA).

## 3. Results

### 3.1. The Characteristics of Patients with CKD Treated and Not Treated with β-Blockers

The patients were divided into two groups: the first group included 23 patients treated with β-adrenoceptor antagonists [β-blockers (+)] and the second group contained 27 patients who did not receive the above medication [β-blockers (−)]. The clinical and biochemical characteristics of the groups are shown in Table 1. Both groups were comparable in terms of age, gender, BP values, index of glomerular filtration rate (eGFR), and stage of their CKD. There were also no differences in the values of the assessed hematological and biochemical parameters between the studied patient groups. The etiology of CKD was similar between the studied groups. Apart from β-blockers, other classes of antihypertensive drugs were prescribed with similar frequency in both study groups. In the β-blockers group, the most commonly prescribed drugs were metoprolol (78%), bisoprolol (13%), and atenolol (9%). 

### 3.2. The Relationship between the Use of β-Blockers and MMP-2 and TIMP-2 Concentrations in the Whole Group of Patients with CKD

**Figure 1:**
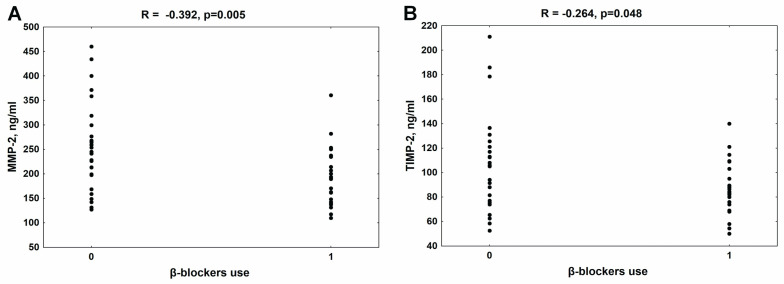
The association between β-blocker use and MMP-2 (**A**) and TIMP-2 levels (**B**) in patients with chronic kidney disease. MMP-2—metalloproteinase-2; TIMP-2—tissue inhibitor of metalloproteinase-2.

In the whole group of patients with CKD, a similar median value of MMP-2 [213.3 (159.0–264.0) ng/mL)] was observed compared to the control group [192.3 (150.8–256.0 ng/mL)]. The median of TIMP-2 concentration in patients was 88.5 (50.0–211.0) ng/mL, while in controls it was 96.5 (90.0–105.5) ng/mL. As presented in Figure 1, the use of β-blockers was significantly associated with lower MMP-2 (Figure 1A) and TIMP-2 concentrations (Figure 1B) in the whole group of patients with CKD. There was no relationship between the MMP-2/TIMP-2 system and the use of other classes of antihypertensive drugs in these patients.

### 3.3. The Effect of β-Blocker Treatment on MMP-2 and TIMP-2 Levels and the MMP-2/TIMP-2 Ratio in Patients with CKD

**Figure 2:**
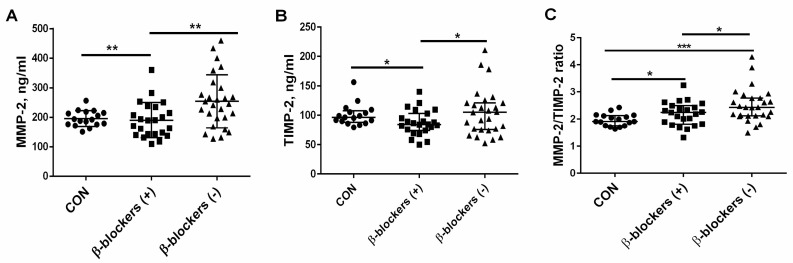
The effect of β-blocker treatment on MMP-2 (**A**), TIMP-2 levels (**B**), and the MMP-2/TIMP-2 ratio (**C**) in patients with chronic kidney disease. The graph illustrates the median values and interquartile range, while the lines represent a comparison between the specified groups. * *p* < 0.05, ** *p* < 0.01, *** *p* < 0.001. MMP-2—metalloproteinase-2; TIMP-2—tissue inhibitor of metalloproteinase-2; β-blockers (+)—the patients treated with β-blockers; β-blockers (−)—the patients untreated with β-blockers.

In the group of CKD patients treated with β-blockers, a similar median value of MMP-2 [189.6 (142.8–234.6) ng/mL)] was observed compared to the controls [192.3 (176.5–214.1 ng/mL)]. The patients not treated with β-blockers had higher values of MMP-2 [245.4 (197.4–299.4) ng/mL] in comparison with the controls (*p* = 0.015), and especially with the β-blockers (+) group (*p* = 0.006); see Figure 2A. The median of TIMP-2 was significantly lower in CKD patients treated with β-blockers [84.5 (74.0–103.0 ng/mL)] than in controls [96.5 (90.0–105.5) ng/mL], *p* = 0.025. The median of TIMP-2 in patients not treated with β-blockers was 105.0 (75.5–121.0) ng/mL, and it was higher than observed in the β-blockers (+) group (*p* = 0.038); see Figure 2B. The MMP-2/TIMP-2 ratio, reflecting an imbalance between MMP-2 and its endogenous inhibitor, was increased both in patients treated with β-blockers (2.19 ± 0.43), and particularly in patients not treated with β-blockers (2.50 ± 0.47) compared to the healthy controls (1.97 ± 0.22), *p* = 0.040 and *p* < 0.001, respectively. The value of this parameter was elevated in the β-blockers (−) group in comparison with the β-blockers (+) group, *p* = 0.045; see Figure 2C.

### 3.4. The Effect of β-Blocker Treatment on Proinflammatory Cytokines and the Marker of Oxidative Stress—Cu/Zn SOD— Levels

**Figure 3:**
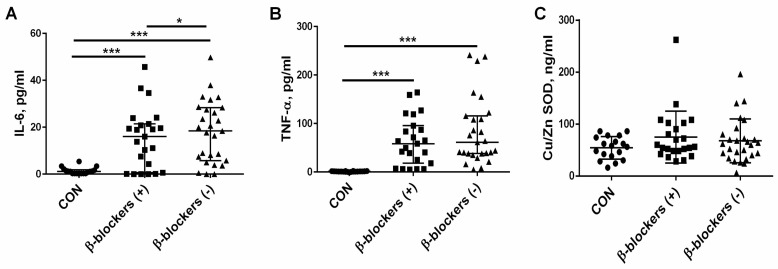
The effect of β-blocker treatment on the two proinflammatory cytokines IL-6 (**A**) and TNF-α (**B**), and the marker of oxidative stress—Cu/Zn SOD— levels (**C**) in patients with chronic kidney disease. The graph illustrates the median values and interquartile range, while the lines represent a comparison between the specified groups. * *p* < 0.05, *** *p* < 0.001. IL-6—interleukin 6; TNF-α—tumor necrosis factor α; Cu/Zn SOD—Cu/Zn superoxide dismutase; β-blockers (+)—the patients treated with β-blockers; β-blockers (−)—the patients untreated with β-blockers.

The concentrations of the proinflammatory cytokines IL-6 and TNF-α were measured in patients with CKD, which were treated or untreated with β-blockers. The median of IL-6 in the controls was 1.1 (1.12–1.76 pg/mL), significantly lower than that observed in CKD patients treated with β-blockers [14.04 (0.61–21.42) pg/mL, *p* = 0.005] or untreated with these drugs [19.94 (5.75–28.64) pg/mL, *p* < 0.001]. The median of IL-6 was reduced in the β-blockers (+) group compared to the β-blockers (−) group, *p* = 0.045; see Figure 3A. The median of TNF-α was significantly higher in the β-blockers (+) group [85.2 (63.5–121.0) pg/mL] and in the β-blockers (−) group [108.5 (69.8–159.3 pg/mL] in comparison with the control group [1.3 (1.12–1.76) pg/mL, both *p* < 0.001]. The median of TNF-α was slightly, but not significantly, increased in patients who did not take β-blockers compared to patients on β-blocker therapy (*p* = 0.406); see Figure 3B. The median level of Cu/Zn SOD, which is a recognized marker of oxidative stress in the population of CKD patients [30,31], was similar in all study groups and amounted to 56 (24–86) ng/mL in the controls, 56 (28–262) ng/mL in the patients treated with β-blockers, and 63 (24–196) ng/mL in the patients who did not take β-blockers (Figure 3C).

### 3.5. The Factors Affecting the MMP-2/TIMP-2 System in the β-Blockers (+) Group and β-Blockers (−) Group

**Figure 4:**
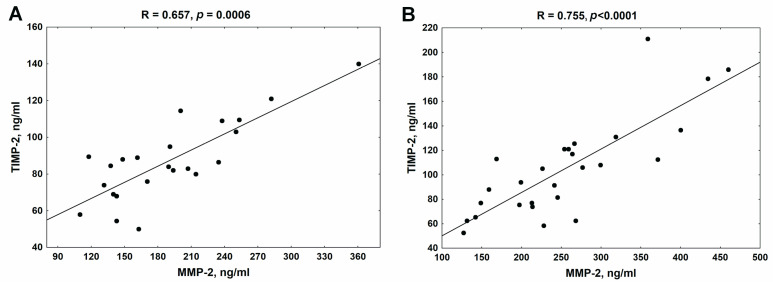
The association between MMP-2 and TIMP-2 in patients with chronic kidney disease treated with β-blockers (**A**) and untreated with β-blockers (**B**). MMP-2—metalloproteinase-2; TIMP-2—tissue inhibitor of metalloproteinase-2.

As shown in Figure 4, there was a strong positive association between MMP-2 and its endogenous inhibitor TIMP-2 in both analyzed patient groups. 

The existing relationships between the MMP-2/TIMP-2 system and the biochemical and clinical parameters of CKD patients both treated and untreated with β-blockers are presented in Table 2. In the group of patients taking β-blockers, the inverse correlation existed between the MMP-2/TIMP-2 system and RBC and hemoglobin concentrations. MMP-2 was inversely associated with albumin and total protein concentrations, whereas TIMP-2 was positively associated with SBP values. Moreover, a positive relation existed between the proinflammatory cytokines IL-6 and TNF-α, and MMP-2; additionally, there was a tendency for a positive correlation between IL-6 and TIMP-2 levels. 

In the group of patients not taking β-blockers, the positive association was between the MMP-2/TIMP-2 system and Cu/Zn SOD levels, whereas the inverse relationship existed between MMP-2 and albumin concentration. The men in this group had elevated MMP-2 values compared to women [263.7 (219.9–338.7) vs. 213.0 (148.8–264.0) ng/mL, *p* = 0.039]. Moreover, the presence of diabetic nephropathy had a significant impact on the MMP-2/TIMP-2 system. A statistically significant increase in MMP-2 concentration was observed in patients with diabetic nephropathy [318.6 (264.0–434.4) ng/mL] compared to people without the above disease [227.1 (168.6–268.2) ng/mL], *p* = 0.019. TIMP-2 concentrations were also significantly higher in patients with diabetic nephropathy [131 (121.0–178.5) ng/mL] compared to those not suffering from the disease [89.8 (74.0–112.5) ng/mL, *p* = 0.007].

The presence of glomerulonephritis (GN) had a statistically significant effect on the reduction of MMP-2 levels both in patients treated with β-blockers [139.8 (117.6–142.8) vs. 192.3 (161.4–237.6) ng/mL, *p* = 0.044] and in patients not taking β-blockers [217.4 (159.0–276.6) vs. 259.2 (226.2–318.6) ng/mL, *p* = 0.036]. Moreover, in the β-blockers (−) group, the occurrence of GN was associated with a decrease in TIMP-2 levels [84.75 (65.5–108.0) ng/mL vs. 121.0 (94.0–131.0 ng/mL, *p* = 0.029)].

### 3.6. The Effect of Calcium Channel Antagonists (CCA) on the MMP-2/TIMP-2 System, Oxidative Status, and Proinflammatory Cytokines in the β-Blockers (+) Group and β-Blockers (−) Group

**Figure 5:**
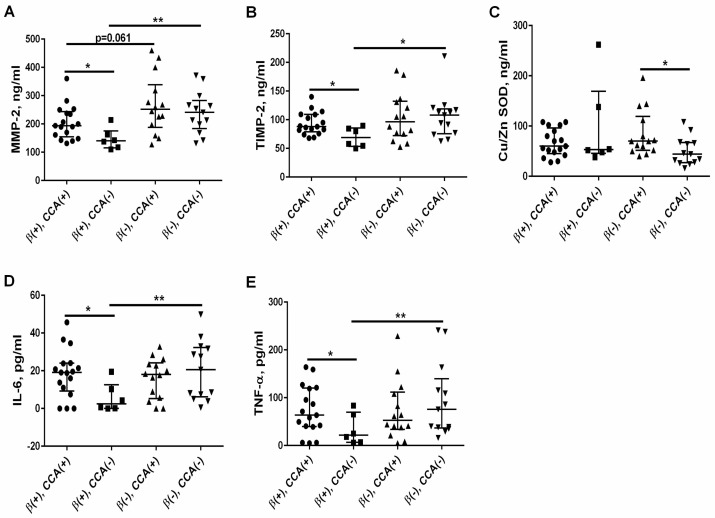
The effect of calcium channel antagonists (CCA) on the levels of MMP-2 (**A**), TIMP-2 (**B**), Cu/Zn SOD (**C**), IL-6 (**D**), and TNF-α (**E**) in patients with CKD both treated and untreated with β-blockers. The graph illustrates the median values and interquartile range, while the lines represent a comparison between the specified groups. * *p* < 0.05, ** *p* < 0.01. MMP-2—metalloproteinase-2; TIMP-2—tissue inhibitor of metalloproteinase-2; Cu/Zn SOD—Cu/Zn superoxide dismutase; IL-6—interleukin 6; TNF-α—tumor necrosis factor α; β(+)—the patients treated with β-blockers; β(−)—the patients untreated with β-blockers; CCA(+)—the patients treated with calcium channel antagonists; CCA(−)—the patients untreated with calcium channel antagonists.

In the β-blockers (+) group, 74% of the patients were also taking CCA, and we noticed that this therapy significantly increased the concentration of MMP-2 [193.8 (161.4–237.6) vs. 140.1 (117.6–162.6) ng/mL, *p* = 0.030] and TIMP-2 [88.0 (82.0–109.0) vs. 69.0 (54.5–84.5) ng/mL, *p* = 0.036]; see Figure 5A,B. IL-6 and TNF-α concentrations were also higher in CCA-treated patients than in CCA-untreated patients [19.44 (11.06–23.92) vs. 2.46 (0.070–10.32) pg/mL, *p* = 0.016] and [63.5 (40.5–119.0) vs. 21.5 (7.0–65.2) pg/mL, *p* = 0.025], respectively; see Figure 5D,E. Meanwhile, CCA therapy did not affect Cu/Zn SOD levels, which were 60 (48–90 ng/mL) in those treated with CCA and 53 (48–138) in those untreated, *p* = 0.888; see Figure 5C.

In order to check whether the observed effect of β-blockers on the above parameters was not due to the difference in the frequency of CCA use in this group, we compared the above parameters among patients not treated with CCA who took or did not take β-blockers. As presented in Figure 5, the patients treated with β-blockers in the absence of CCA had significantly lower levels of MMP-2, TIMP-2, IL-6, and TNF-α than those who were not treated with β-blockers. Interestingly, the effect of β-blockers on these parameters was more evident in the absence of CCA than in the simultaneous use of β-blockers and CCA. Meanwhile, in the β-blockers (−) group, the patients treated with CCA had significantly higher Cu/Zn SOD levels than those not treated with this class of drugs (Figure 5C).

### 3.7. Variables Independently Predicting the MMP-2/TIMP-2 System in the β-Blockers Group (+) and β-Blockers (−) Group

To determine which variables could independently affect the MMP-2/TIMP-2 system in the β-blockers (+) and β-blockers (−) groups, the multiple regression analysis was performed based on the results of Spearman’s rank correlation analysis and quasi-Newton and Rosenbrock’s regression analysis. As presented in Table 3, the low albumin concentration followed by reduced RBC independently and significantly predicted elevated MMP-2 levels in patients treated with β-blockers. In turn, the high MMP-2 levels and elevated SBP values independently affected TIMP-2 concentration in this group.

In the group of patients not taking β-blockers, the high Cu/Zn SOD and low albumin levels were found to be significant independent factors affecting MMP-2 concentration. In the case of TIMP-2, MMP-2 levels and the presence of GN turned out to be independent factors influencing the values of this parameter (Table 4).

## 4. Discussion

In the present study, for the first time, we showed that the use of β-blockers reduces serum levels of MMP-2 and its endogenous inhibitor TIMP-2 in patients with CKD on conservative treatment. The above effect was associated with a simultaneous decrease in IL-6, which is one of the factors inducing MMP-2 gene expression [32,33]. Moreover, the results of our study showed that the MMP-2/TIMP-2 system was determined by distinct clinical and biochemical variables in both analyzed groups.

Previously, we observed the increased values of MMP-2 and TIMP-2 in CKD patients undergoing hemodialysis or peritoneal dialysis therapy [20,21,22,23] but not on conservative treatment [20]. In the present study, the use of β-blockers in patients with CKD was associated with the normalization of MMP-2 levels, as well as with the reduction in TIMP-2 concentrations below values observed in controls. Contrary to the β-blockers (+) group, the values of MMP-2 and TIMP-2 remained significantly elevated in CKD patients not taking β-blockers. Until now, no studies have examined the effect of β-blockers on the MMP-2/TIMP-2 system in patients with CKD. However, the scarce experimental studies suggest that some β-blockers, like nebivolol or carvedilol, can attenuate the level and expression of MMPs [13,14,15,16], and our results are in line with previously mentioned observations. The MMP-2/TIMP-2 ratio, reflecting the functional balance between this metalloproteinase and its endogenous inhibitor [34], was only slightly increased in the β-blockers (+) group, whereas it was considerably higher in the β-blockers (−) group compared to healthy subjects. This is a clinically relevant finding, as the imbalance between MMP-2 and TIMP-2, favoring this metalloproteinase, was associated with excessive substrate turnover and can play an important role in pathological tissue remodeling [19,34]. Indeed, we reported the association of the MMP-2/TIMP-2 system with carotid atherosclerosis and cardiovascular risk in patients with severe CKD on dialysotherapy [21,22,23]. Similarly, Kobusiak-Prokopowicz et al. [35] found that MMP-2 levels were positively correlated both with ejection fraction and the degree of renal failure in patients with heart failure and CKD. Moreover, higher MMP-2 levels were observed after acute myocardial infarction, and they were associated with larger left ventricular volumes [36]. On the other hand, the involvement of MMP-2 and TIMP-2 in kidney disease progression has been demonstrated [37,38]. The above findings indicate that both kidney disease and CVD may predispose to an increase in the MMP-2/TIMP-2 system. Therefore, our results suggest that the use of β-blockers, resulting in a reduction in MMP-2/TIMP-2 imbalance, could provide benefits by attenuating both CKD progression and pathological vascular wall remodeling.

In the next step of the current research, we have tried to find the possible mechanism by which β-blockers could affect the MMP-2/TIMP-2 system. It is well known that MMP-2 synthesis may be regulated by different factors, like proinflammatory cytokines or oxidative stress [39,40], and our previous studies demonstrated such mechanisms in non-dialyzed and dialyzed patients with CKD [20,21,22,23]. Herein, we observed that β-blocker treatment repressed the production of proinflammatory cytokines. This is in agreement with the results of previous studies, demonstrating that β-blockers can down-regulate inflammatory mediators—TNF-α and IL-6—and reduce the inflammatory response both in clinical and experimental conditions [8,9,10,41,42]. It has been proved that MMP-2 expression can be enhanced by inflammatory cytokines and that IL-6, which is a pleiotropic cytokine involved in inflammation, hematopoiesis, vascular permeability, and tissue injury [43], may be a strong inducer of MMP-2 [32,33]. On the other hand, MMP-2 can be induced by oxidative stress [40], which, in turn, may result from β1-adrenoceptor activation [44]. It has been shown that transgenic mice overexpressing β1-adrenoceptors show increased MMP-2 levels and enhanced cardiac remodeling [45]. Rizzi et al. [13] demonstrated that β1 blockers—nebivolol and metoprolol—exerted antioxidant effects, reduced MMP-2 activity, and improved cardiac hypertrophy in a rat two-kidney one-clip (2K1C) hypertension model. However, we did not observe the effect of β-blocker use on the values of Cu/Zn SOD, a marker of oxidative stress in the CKD population recognized by us and others [30,31]. Although we cannot exclude the effect of β-blockers on other markers of oxidative status, our results suggest that the mechanism related to the attenuation of oxidative stress is not likely to be responsible for the MMP-2 reduction shown here. This discrepancy may be due to the fact that our patients were treated with β1-selective blockers (mainly metoprolol), whereas in the previous experimental studies, the “third generation” of β-blockers was used (carvedilol, nebivolol), which are considered to be more effective in the reduction in oxidative stress and MMP-2 activation than classical cardioselective drugs [46,47].

The strong and positive association observed between MMP-2 and TIMP-2 levels both in univariate (Figure 4) as well as in multiple regression analysis (Table 3 and Table 4) in both studied groups indicated that TIMP-2 levels are primarily regulated by alteration in the concentration of MMP-2, and this is in line with the results obtained previously in peritoneally dialyzed patients [23]. In the group of patients undergoing β-blocker therapy, we noticed inverse associations between MMP-2 concentration and RBC, hemoglobin, total protein, and albumin levels. Moreover, the low albumin and reduced RBC turned out to be the independent determinants of MMP-2 levels (Table 3). Meanwhile, a positive relationship existed between MMP-2 and proinflammatory cytokines. Inflammation can interact with the hematopoietic system, inhibiting the maturation of erythroid progenitor cells or increasing erythrocyte destruction [48]. Inflammation may also contribute to malnutrition, leading to a decrease in total protein and albumin levels in the CKD population [49]. Thus, the associations of MMP-2 mentioned above probably, in an indirect manner, reflect the impact of inflammation on this metalloproteinase level, as has been previously shown by us [20]. 

In the β-blockers (−) group, the alterations in the MMP-2/TIMP-2 system were closely related to the oxidative stress marker—Cu/Zn SOD—and this is in line with our previous reports [21,22,23] and others [31,40]. Moreover, the MMP-2/TIMP-2 system depends on the etiology of CKD. In this group, we showed a significant increase in the concentration of MMP-2 and TIMP-2 in diabetic nephropathy, which is consistent with the results of previous studies [50,51]. On the contrary, while the cause of CKD was glomerulonephritis, decreased concentrations of this metalloproteinase were observed in both studied groups, whereas the reduction in TIMP-2 was seen only in the β-blockers (−) group. Other researchers have also demonstrated an MMP/TIMP imbalance in glomerulopathies, which depends on the type of this disease [52]. Finally, multiple regression analysis identified an increased oxidative stress marker and low albumin level as independent determinants of MMP-2 in the β-blockers (−) group.

Interestingly, patients from the β-blockers (+) group, which simultaneously obtained calcium channel antagonists (CCA), presented higher MMP-2, TIMP-2, IL-6, and TNF-α levels than those not treated with this class of drug. This was unexpected, as some previous experimental studies showed that CCA can attenuate inflammation, oxidative stress, and MMP-2 activity [53,54,55]. However, clinical studies have shown that in patients with essential hypertension, MMP-2 levels were raised after 6 months of treatment with felodipine or amlodipine [56,57]. To establish the “pure” effect of β-blockers therapy on these parameters, we compared them in groups of patients treated/or untreated with β-blockers who were not taking CCA at the same time. In these conditions, the MMP-2/TIMP-2 system and proinflammatory cytokines were even further reduced in patients treated with β-blockers than in those untreated with these drugs. On the other hand, the patients treated with CCA had significantly higher oxidative status than those not treated with this class of drugs in the absence of β-blockers, and there was no difference in the oxidative status between β-blockers (+) and β-blockers (−) group. The above results suggest that simultaneous therapy with CCA could interfere with the effects of β-blockers on the attenuation of inflammation, oxidative stress, and reduction in the MMP-2/TIMP-2 system. To our knowledge, the association between CCA use and the studied parameters is presented for the first time in CKD patients treated with β-blockers, and it is difficult to interpret at the moment. Further investigations are required to verify these results.

### Strengths and Limitations

The main limitation of the study is our relatively small study groups and the use of heterogeneous antihypertensive medication across the study groups. Moreover, owing to the lack of data, it is unknown in what doses and how long the patients have been using β-blockers or other antihypertensive drugs. On the other hand, the baseline characteristics of the β-blockers (+) and the β-blockers (−) group were well balanced, limiting the potential impact of these co-variables on the studied parameters. 

## 5. Conclusions

Our study is the first to show that the use of β-blockers was associated with a reduction in MMP-2 level, TIMP-2 level, and the MMP-2/TIMP-2 ratio in patients with CKD on conservative treatment. Mechanistically, this action of β-blockers was related to the decrease in proinflammatory cytokines IL-6 and TNF-α, without an effect on the oxidative stress marker of these patients. The use of calcium channel antagonists together with β-blockers seems to interfere with this beneficial effect of β-blockers; however, the indicated observation needs confirmation in further studies. As the MMP-2/TIMP-2 system contributes to the pathogenesis of atherosclerosis, cardiovascular disease, and the progression of CKD, our findings suggest an underlying pharmacological rationale for the use of β-blockers in clinical practice to reduce inflammation and abnormal vascular remodeling, which predispose the patients with CKD to increased cardiovascular events.

## Figures and Tables

**Table 1:** Basic clinical and biochemical parameters of patients with chronic kidney disease (CKD) treated with β-blockers [β-blockers (+)] and not treated with β-blockers [β-blockers (−)].

	β-Blockers (+), *n* = 23	β-Blockers (−), *n* = 27	*p* Value
Age, years	53.43 ± 12.70	53.48 ± 17.74	0.897
Male sex, %	57	59	0.886
eGFR, mL/min/1.73 m^2^	17.5 (9.7–42.6)	18.7 (12.8–33.8)	0.535
CKD, stage 1, %	0	4	0.332
CKD, stage 2, %	17	15	0.849
CKD, stage 3, %	17	19	0.856
CKD, stage 4, %	17	19	0.856
CKD, stage 5, %	48	42	0.673
SBP, mm Hg	136.90 ± 11.23	134.23 ± 14.26	0.471
DBP, mm Hg	88.57 ± 7.27	84.62 ± 7.20	0.695
Heart rate, bpm	73.3 ± 8.9	68.8 ± 9.5	0.562
RBC, 10^12^/L	3.63 ± 0.72	3.45 ± 0.57	0.334
Hemoglobin, g/dL	11.13 ± 2.35	10.70 ± 1.98	0.490
WBC, 10^9^/L	6.41 ± 1.95	6.49 ± 2.62	0.560
Glucose, mg/dL	90.0 (81.0–100.0)	93.5 (85.0–117.0)	0.474
Albumin, g/dL	3.12 ± 0.71	3.07 ± 0.77	0.814
Total protein, g/dL	6.10 ± 1.22	6.10 ± 1.17	0.999
Total cholesterol, mg/dL	208.0 (175.0–223.0)	190.0 (170.0–219.0)	0.338
Triglycerides, mg/dL	170 (115–265)	152 (80–291)	0.448
Cardiovascular disease, %	48	37	0.432
Smokers, %	9	19	0.316
Diabetic nephropathy, %	37	30	0.652
Glomerulonephritis, %	37	47	0.475
Polycystic kidney disease, %	17	19	0.854
Hypertensive nephropathy, %	9	4	0.234
Diuretics, %	26	15	0.166
Converting enzyme inhibitors/sartans %	65	52	0.176
Calcium channel antagonists, %	74	52	0.110
α-adrenoceptor antagonists, %	13	4	0.123
Statin, %	9	19	0.316

Data are presented as means ± standard deviations or medians (interquartile ranges) for continuous variables and % for categorical variables. SBP—systolic blood pressure; DBP—diastolic blood pressure; RBC—red blood cell count; WBC—white blood cell count.

**Table 2:** Relationships between the MMP-2/TIMP-2 system and the biochemical parameters and clinical characteristics of patients with chronic kidney disease (CKD) treated with β-blockers [β-blockers (+)] and not treated with β-blockers [β-blockers (−)].

	β-Blockers (+)		β-Blockers (−)	
	MMP-2	TIMP-2	MMP-2	TIMP-2
Sex, male = 1	R = 0.031 NS	R = 0.008 NS	χ^2^ = 4.518 *p* = 0.034	χ^2^ = 2.706 NS
SBP	R = 0.163 NS	R = 0.463 *p* = 0.035	R = 0.180 NS	R = 0.150 NS
RBC	R = −0.500 *p* = 0.015	R = −0.509 *p* = 0.013	R = −0.142 NS	R = −0.075 NS
Hemoglobin	R = −0.510 *p* = 0.013	R = −0.556 *p* = 0.006	R = −0.102 NS	R = −0.046 NS
Interleukin 6	R = 0.458 *p* = 0.028	R = 0.390 *p* = 0.066	R = 0.101 NS	R = 0.061 NS
TNF-α	R = 0.419 *p* = 0.046	R = 0.230 NS	R = 0.042 NS	R = 0.062 NS
Cu/Zn SOD	R = −0.066 NS	R = 0.077 NS	R = 0.382 *p* = 0.049	R = 0.549 *p* = 0.003
Albumin	R = −0.586 *p* = 0.002	R = −0.290 NS	R = −0.471 *p* = 0.030	R = −0.300 NS
Total protein	R = −0.544 *p* = 0.008	R = −0.082 NS	R = −0.273 NS	R = −0.056 NS
Diabetic nephropathy	χ^2^ = 0.016 NS	χ^2^ = 0.020 NS	χ^2^ = 6.497 *p* = 0.011	χ^2^ = 6.618 *p* = 0.010
Glomerulonephritis	χ^2^ = 4.422 *p* = 0.035	χ^2^ = 2.782 NS	χ^2^ = 6.253 *p* = 0.010	χ^2^ = 6.367 *p* = 0.012
Calcium channel antagonists	χ^2^ = 6.199 *p* = 0.013	χ^2^ = 7.475 *p* = 0.006	χ^2^ = 0.514 NS	χ^2^ = 0.019 NS

Correlations were determined using Spearman’s rank correlation analysis and quasi-Newton and Rosenbrock’s regression analysis. SBP—systolic blood pressure; RBC—red blood count; TNF-α—tumor necrosis factor-α; Cu/Zn SOD—Cu/Zn superoxide dismutase.

**Table 3:** Variables independently affecting the MMP-2/TIMP-2 system in the β-blockers (+) group.

	Independent Variable	Regression Coefficient (β)	Standard Error	*p* Values
MMP-2	albumin	−0.553	0.181	<0.001
	RBC	−0.512	0.178	0.016
TIMP-2 *	MMP-2	0.740	0.132	<0.001
	SBP	0.297	0.132	0.038

Multiple *r* for variables in the model = 0.725 (* 0.829), multiple *r*^2^ = 0.526 (* 0.688), and adjusted *r*^2^ = 0.453 (* 0.653); all *p* < 0.001. MMP-2—metalloproteinase 2; TIMP-2—type 2 metalloproteinase inhibitor; RBC—red blood cells; SBP—systolic blood pressure.

**Table 4:** Variables independently affecting the MMP-2/TIMP-2 system in the β-blockers (−) group.

	Independent Variable	Regression Coefficient (β)	Standard Error	*p* Values
MMP-2	Cu/Zn SOD	0.734	0.208	0.005
	albumin	−0.682	0.242	0.018
TIMP-2 *	MMP-2	0.739	0.113	<0.001
	glomerulonephritis	−0.264	0.112	0.028

Multiple *r* for variables in the model = 0.827 (* 0.846), multiple *r*^2^ = 0.684 (* 0.715), and adjusted *r*^2^ = 0.589 (* 0.691); *p* = 0.007 and *p* < 0.001, respectively. MMP-2—metalloproteinase 2; TIMP-2—type 2 metalloproteinase inhibitor; Cu/Zn SOD—Cu/Zn superoxide dismutase.

## Data Availability

The data presented in this study are available upon request from the corresponding author.
